# High Throughput Screening of CMAS Corrosion‐Resistant RETaO_4_ Based on Lamination Method

**DOI:** 10.1002/advs.202412717

**Published:** 2025-02-17

**Authors:** Zhilin Tian, Zhilin Chen, Shuping Wen, Wenxia Zhao, Liya Zheng, Bin Li

**Affiliations:** ^1^ School of Materials Shenzhen Campus of Sun Yat‐sen University Shenzhen 518107 China; ^2^ Guangdong‐Hong Kong Joint Laboratory of Modern Surface Engineering Technology Guangdong Provincial Key Laboratory of Modern Surface Engineering Technology Institute of New Materials Guangdong Academy of Sciences Guangzhou 510650 China; ^3^ Instrumental analysis and research center Sun Yat‐sen University Guangzhou 510275 China

**Keywords:** CMAS corrosion, high throughput, rare earth ionic radii, rare earth tantalates, thermal barrier coatings

## Abstract

Rare earth tantalates (RETaO_4_), known for their exceptional thermomechanical properties, are promising candidates for next‐generation thermal barrier coatings (TBCs). However, the role of rare earth (RE) species in the CMAS (calcium‐magnesium‐aluminosilicate) corrosion behavior and mechanisms of RETaO_4_ remains unclear, hindering their design and application as TBCs. This study employs a high‐throughput approach to systematically investigate the CMAS corrosion mechanisms of RETaO_4_ (RE = Nd, Sm, Eu, Gd, Dy, Ho, Y, and Er) at 1300 °C. Precise analysis of the microstructure and composition reveal that the primary corrosion products are (Ca_2‐x_RE_x_)(Ta_2‐y‐z_Mg_y_Al_z_)O_7_ solid solutions, along with minor amounts of Ca_2_RE_8_(SiO_4_)_6_O_2_ apatite. These corrosion products are observed both in the recession layer and at grain boundaries. The CMAS infiltration depth of RETaO_4_ increases with the RE ionic radius. First‐principles calculations indicate that the formation enthalpy of corrosion products becomes more exothermic as the RE ionic radius increases, promoting the formation of corrosion products. Additionally, the wetting behavior of liquid CMAS on RETaO_4_ at high temperatures supports that RETaO_4_ with smaller RE ionic radius present better corrosion resistance. These findings provide insights into the influence of RE species on the CMAS corrosion behavior of RETaO_4_, offering guidelines for the rapid screening of CMAS‐resistant TBC materials.

## Graphical abstract

This work employs a high‐throughput approach to investigate the CMAS corrosion behavior of RETaO_4_ at 1300 °C. Corrosion products are found in both the recession layer and at grain boundaries. The CMAS infiltration depth shows a positive correlation with the RE ionic radius, attributed to their influence on the formation enthalpy of the corrosion products.

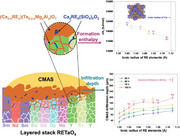

## 1 Introduction

The development of gas turbine engines is primarily driven by the need for a high thrust‐to‐weight ratio and increased efficiency, leading to a continuous rise in the operating temperatures of critical components, especially the superalloy turbine blades.^[^ ^1^, ^2^ ^]^ As a result, service temperatures may approach or exceed the temperature limits of superalloys.^[^ ^3^ ^]^ Traditional thermal barrier coatings, such as yttria‐stabilized zirconia (YSZ), are prone to phase transitions at temperatures above 1200 °C, which can result in TBC spallation.^[^ ^4^, ^5^, ^6^, ^7^, ^8^ ^]^

RETaO_4_ ceramics have garnered significant attention as potential next‐generation TBC materials due to their advantageous properties, including low thermal conductivity ((1.4∼2.1) W·m⁻¹·K⁻¹ at 900 °C), high‐temperature resistance, compatible thermal expansion coefficients, and ferroelastic toughening mechanisms.^[^ ^9^, ^10^, ^11^, ^12^, ^13^, ^14^ ^]^ In addition to thermomechanical properties, corrosion resistance is also a key criterion for evaluating TBC materials.^[^ ^15^, ^16^ ^]^ High‐temperature environments can expose TBCs to particulates such as sand, fly ash, and volcanic ash, leading to severe corrosion and premature failure.^[^ ^17^, ^18^, ^19^, ^20^, ^21^, ^22^ ^]^ When the temperature exceeds about 1240 °C, these particulates form calcium‐magnesium‐aluminosilicate (CMAS) glassy melts, which deposit on TBC surface, progressively dissolving the coating and precipitating corrosion products.^[^ ^23^, ^24^, ^25^, ^26^ ^]^ Current studies on the CMAS corrosion of RETaO_4_ have produced conflicting findings regarding corrosion products, reaction mechanisms, and CMAS infiltration pathways.^[^ ^27^, ^28^, ^29^, ^30^, ^31^, ^32^, ^33^ ^]^ For instance, Ye et al. instance that Ca_2_Ta_2_O_7_, Y_2_Si_2_O_7_, and M’‐YTaO_4_ are the primary reaction products in YTaO_4_ after CMAS corrosion.^[^ ^27^, ^32^ ^]^ In contrast Zhao et al. propose that M’‐YTaO_4_ and Ca_0.135_Y_0.449_Ta_0.081_Si_0.336_O_1.683_ apatite are the main products.^[^ ^33^ ^]^ Our recent research has shown, through precise compositional analysis, that the dominant reaction product of YTaO_4_ interacting with CMAS is a complex solid solution, (Ca_2‐x_Y_x_)(Ta_2‐y‐z_Mg_y_Al_z_)O_7_.^[^ ^29^ ^]^ Furthermore, grain boundary infiltration of CMAS was discovered in YTaO_4_ for the first time.^[^ ^29^ ^]^ The discrepancies in the conclusions of various studies primarily arise from differences in experimental conditions and methodologies. Moreover, the broad variety of RE elements presents challenges for traditional techniques to efficiently and systematically evaluate the influence of RE element species on the CMAS corrosion behavior of RETaO_4_. These limitations have significantly hindered the screening and practical application of CMAS‐resistant RETaO_4_. Consequently, it is essential to employ a high‐throughput method that enables systematic CMAS corrosion tests on a series of RETaO_4_ compounds under consistent conditions. This approach allows for the rapid and precise comparison of the CMAS corrosion behaviors of RETaO_4_ compounds with different RE elements, which is critical for advancing material selection and application.

In this work, a high‐throughput method based on a layered configuration was employed to systematically investigate the CMAS corrosion behavior of a series of RETaO_4_ compounds at 1300 °C under uniform experimental conditions. Cross‐sectional analyses of the corroded samples allow for direct and efficient comparisons of CMAS infiltration pathways and depths among the RETaO_4_. Through precise analysis of the microstructure and composition of corrosion products, the specific mechanism of CMAS corrosion has been further elucidated, and the influence of RE species on corrosion resistance was clarified. RETaO_4_ compounds exhibiting exceptional CMAS resistance were efficiently identified, advancing the development and application of RETaO_4_‐based TBCs. This study also provides a highly efficient approach for the rapid screening of CMAS‐resistant materials for TBCs.

## 2 Results

### 2.1 High‐Throughput Investigation of CMAS Corrosion Behavior in RETaO_4_

**Figure 1:**
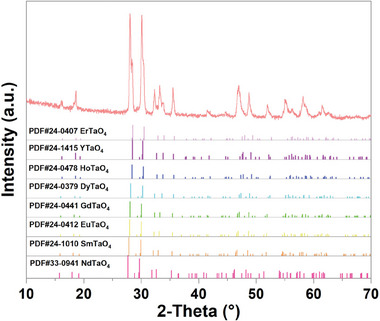
XRD pattern of the as‐prepared layered stack RETaO_4_ ceramic.

The X‐ray diffraction (XRD) pattern of the as‐prepared layered stack RETaO_4_ ceramic is shown in **Figure** **1**. The diffraction peaks of the layered stack ceramic were consistent with the standard XRD spectra of RETaO_4_, confirming the presence of a pure RETaO_4_ phase without impurities. However, the broadening and splitting of the diffraction peaks suggest that multiple RETaO_4_ phases coexist in the sample, rather than forming a single‐phase solid solution.

**Figure 2:**
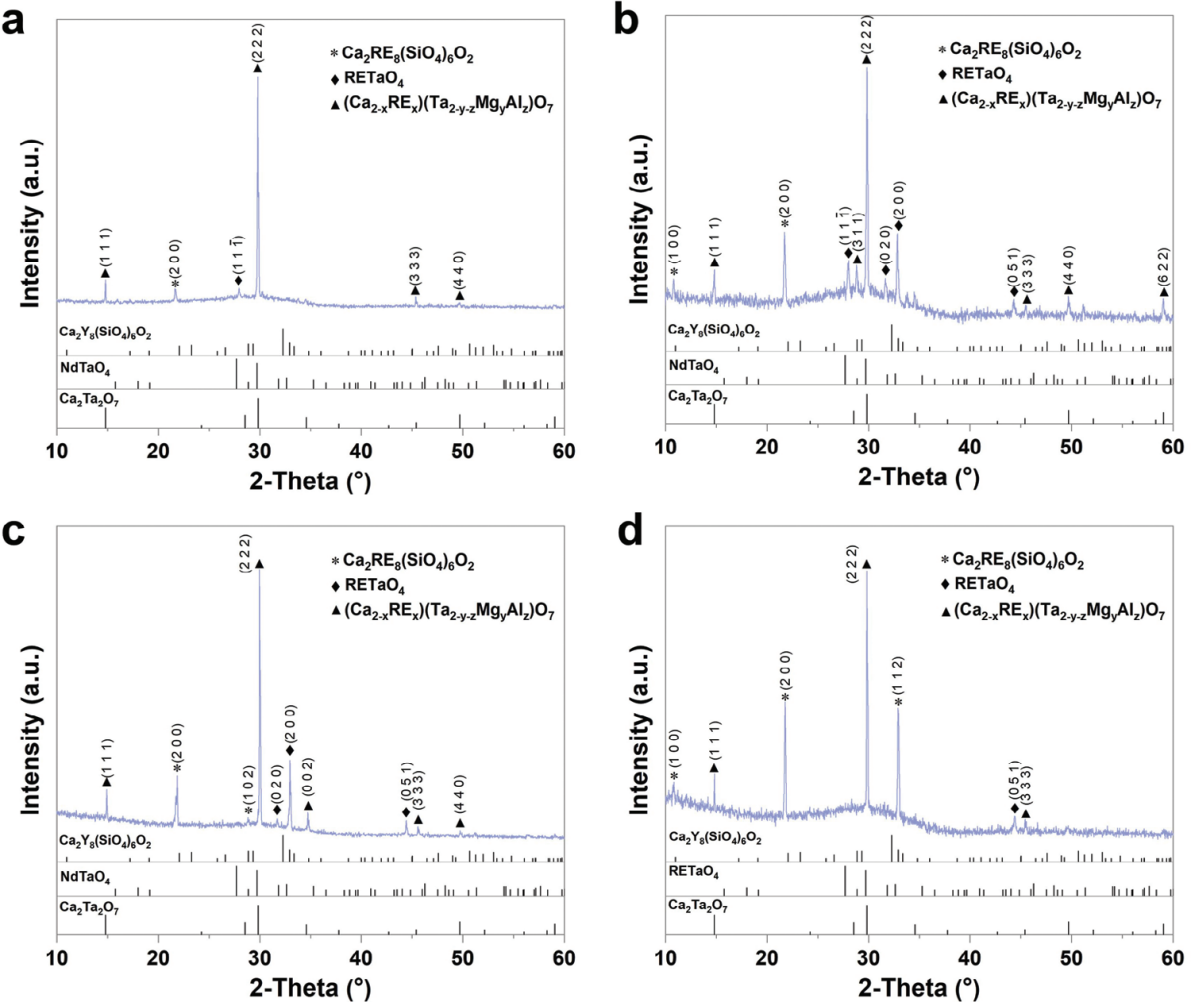
XRD patterns of layered stack RETaO_4_ ceramics after CMAS corrosion at 1300 °C. a) 25, b) 50, c) 75, and d) 100 h.

To investigate the reaction mechanisms during corrosion, XRD analysis was conducted on samples after exposure to CMAS at 1300 °C for 25, 50, 75, and 100 h, as illustrated in **Figure** **2**. Over time, the diffraction peaks of the primary corrosion products align with those of Ca_2_Ta_2_O_7_. However, reports suggest that Ca and Ta in Ca_2_Ta_2_O_7_ can be substituted to form complex solid solutions, such as Ca_0.8_Ti_1.35_Zr_1.3_Th_0.15_Al_0.4_O_7_, Ca_1.92_Ta_1.82_Nd_0.08_Zr_0.08_O_7_,^[^ ^34^ ^]^ and Ca_1.80_Ta_1.80_Sm_0.24_Ti_0.17_O_7_.^[^ ^35^ ^]^ Combined with electron probe microanalyzer (EPMA) analysis (in the subsequent analysis) and our previous work,^[^ ^29^ ^]^ it can be proven that the main reaction product is (Ca_2‐x_RE_x_)(Ta_2‐y‐z_Mg_y_Al_z_)O_7_ solid solutions. Meanwhile, a small amount of Ca_2_RE_8_(SiO_4_)_6_O_2_ apatite was also detected after CMAS corrosion, as shown in Figure 2. In addition, the diffraction peak corresponding to the (2 2 2) plane of the (Ca_2‐x_RE_x_)(Ta_2‐y‐z_Mg_y_Al_z_)O_7_ solid solution is prominent, indicating the existence of a preferred orientation for this crystal plane.

**Figure 3:**
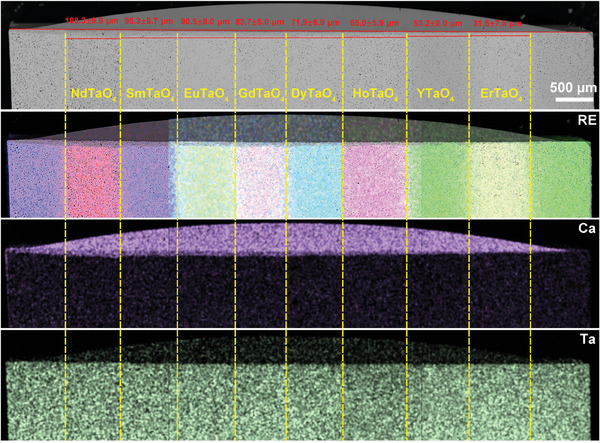
Cross‐sectional morphologies and energy dispersive spectroscopy (EDS) mapping of layered stack RETaO_4_ ceramic after CMAS corrosion at 1300 °C for 25 h.

**Figure 4:**
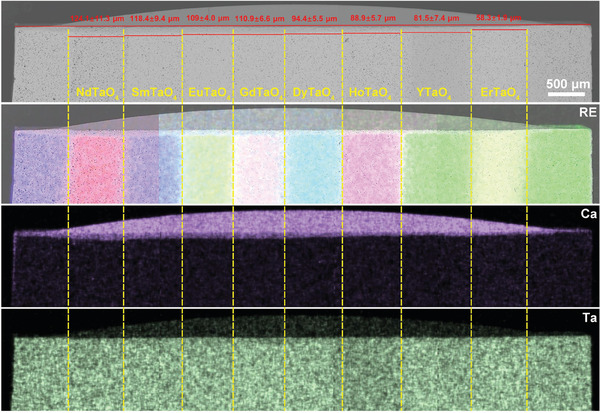
Cross‐sectional morphologies and EDS mapping of layered stack RETaO_4_ ceramic after CMAS corrosion at 1300 °C for 50 h.

**Figure 5:**
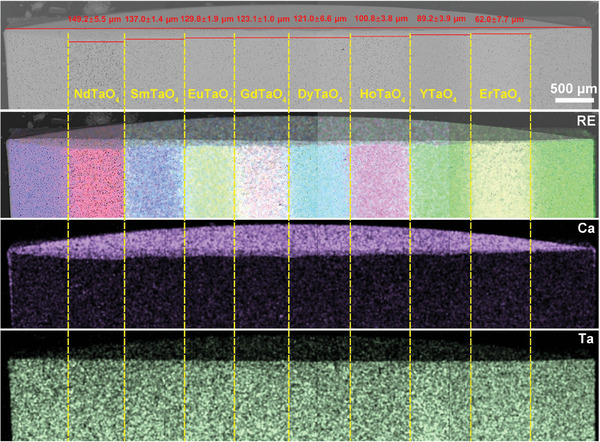
Cross‐sectional morphologies and EDS mapping of layered stack RETaO_4_ ceramic after CMAS corrosion at 1300 °C for 75 h.

**Figure 6:**
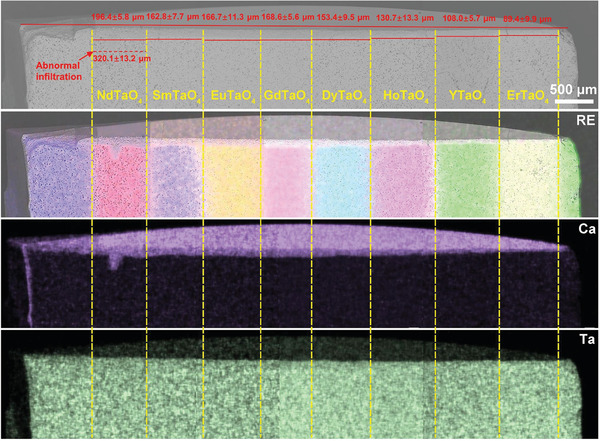
Cross‐sectional morphologies and EDS mapping of layered stack RETaO_4_ ceramic after CMAS corrosion at 1300 °C for 100 h.

Layered stack ceramics allow for a visual comparison of the corrosion behavior of different RETaO_4_ under the identical CMAS corrosion conditions. **Figures** 3, 4, 5, 6 exhibit the overall cross‐sectional morphology and elemental distribution of the samples after CMAS corrosion at 1300 °C for 25, 50, 75, and 100 h, respectively. Each layer (ranging from NdTaO_4_ to ErTaO_4_) of the RETaO_4_ was fully covered with sufficient CMAS molten salt, ensuring that the corrosion reaction proceeded. The additional SmTaO_4_ or YTaO_4_ layers at both ends, not covered by CMAS, served as benchmarks for measuring CMAS infiltration depth. The distribution of RE elements at the boundaries of each layer remained distinct, with no evident diffusion between adjacent layers, confirming that the CMAS corrosion in one layer did not affect adjacent layers. The CMAS infiltration depth in each layer can be measured through cross‐sectional morphology by the mapping of Ca and RE elements. After different corrosion durations, the CMAS infiltration depth of RETaO_4_ gradually decreases with the reduction of the radius of RE^3+^. For example, after 50 h of corrosion, the CMAS infiltration depth ranged from 124.1 ± 11.3 µm in NdTaO_4_ to 58.3 ± 1.9 µm in ErTaO_4_. With increased corrosion duration, the infiltration depth grew significantly, with ErTaO_4_ showing the best resistance to CMAS corrosion. When the corrosion duration is 25 h, the CMAS infiltration depth is 31.5 ± 7.8 µm and even after 100 h interactions, the CMAS infiltration depth is only 89.4 ± 9.9 µm. In contrast, NdTaO_4_ experienced more intense CMAS corrosion, with an infiltration depth of 108.3 ± 9.8 µm after 25 h, increasing to 196.4 ± 5.8 µm after 100 h of corrosion. Furthermore, as shown in Figure 6, defects in NdTaO_4_, such as large pores, led to localized severe CMAS infiltration, reaching a depth of 320.1 ± 13.2 µm.

**Figure 7:**
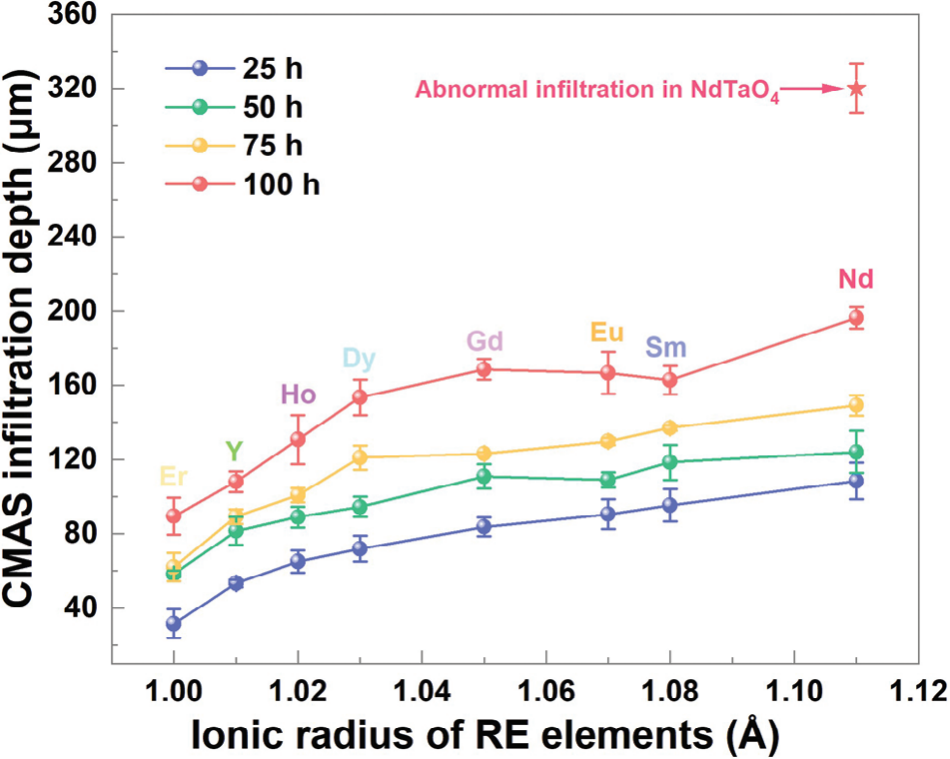
Relationship between CMAS infiltration depth and RE ionic radius in layered stack RETaO_4_ ceramics.

The CMAS corrosion resistance of some thermal and environmental barrier coatings is usually correlated with the ionic radius of the incorporated RE elements.^[^ ^36^, ^37^ ^]^ The relationship between the CMAS infiltration depth and the RE ionic radius in each layer of stacked RETaO_4_ samples is summarized in **Figure** **7**. With prolonged exposure, the CMAS infiltration depth of each layer shows an increasing trend with the larger ionic radius of RE elements. RETaO_4_ compounds such as ErTaO_4_, YTaO_4_, and HoTaO_4_ exhibited excellent CMAS resistance, displaying shallow CMAS infiltration depths. Conversely, RETaO_4_ samples containing Dy, Gd, Eu, and Sm were more severely affected by CMAS corrosion, with infiltration depths reaching approximately 160 µm after 100 h. The CMAS infiltration depths of EuTaO_4_ and SmTaO_4_ are slightly lower than that of GdTaO_4_. This is mainly attribute to the gradual thickening and densification of the reaction layer over time, which effectively impedes further CMAS penetration. NdTaO_4_ showed the highest susceptibility to CMAS, with a marked increase in infiltration depth after 100 h.

### 2.2 CMAS Grain Boundary Corrosion in RETaO_4_

**Figure 8:**
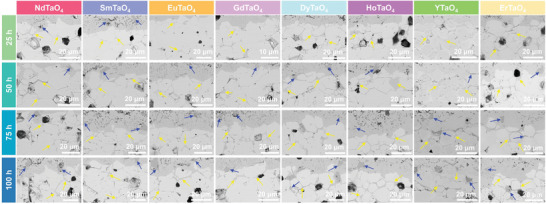
High‐magnification SEM images of reaction front in layered stack RETaO_4_ ceramics after CMAS corrosion at 1300 °C for 25, 50, 75, and 100 h.

At high temperatures, some thermal and environmental barrier coating materials not only dissolve in CMAS molten salts and precipitate corrosion products but also experience severe infiltration of CMAS into their grain boundaries, such as YSZ,^[^ ^38^ ^]^ La_2_Zr_2_O_7_,^[^ ^39^ ^]^ and RE_2_Si_2_O_7_.^[^ ^40^, ^41^, ^42^ ^]^ Our recent work demonstrated CMAS grain boundary corrosion in YTaO_4_,^[^ ^29^ ^]^ but it remains unclear whether other RETaO_4_ exhibit similar behavior. **Figure** **8** presents high‐magnification SEM images of the reaction front after CMAS corrosion. Significant grain boundary corrosion (highlighted by yellow arrows) was evident in all RETaO_4_ compounds. Additionally, a minor amount of another product, marked by blue arrows, was observed in the reaction layer and grain boundaries.

**Figure 9:**
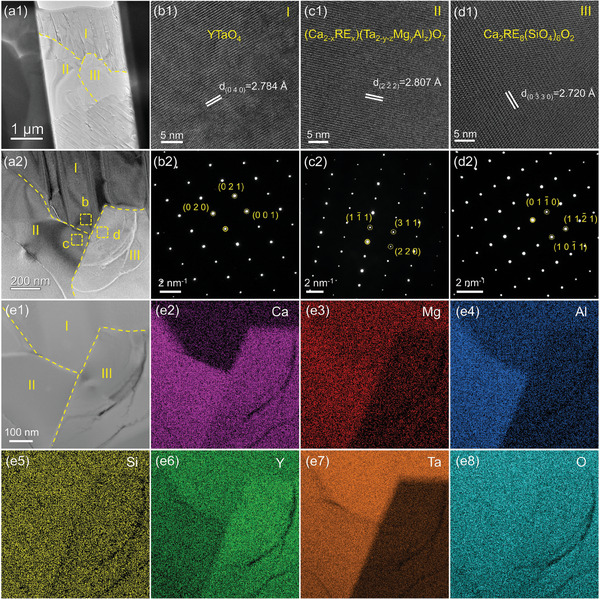
a) TEM images of the reaction front in the YTaO_4_ after CMAS corrosion at 1300 °C for 50 h; b–d) HRTEM and SAED images of the corresponding regions in (a); e) STEM image and corresponding EDS mapping.

**Figure 10:**
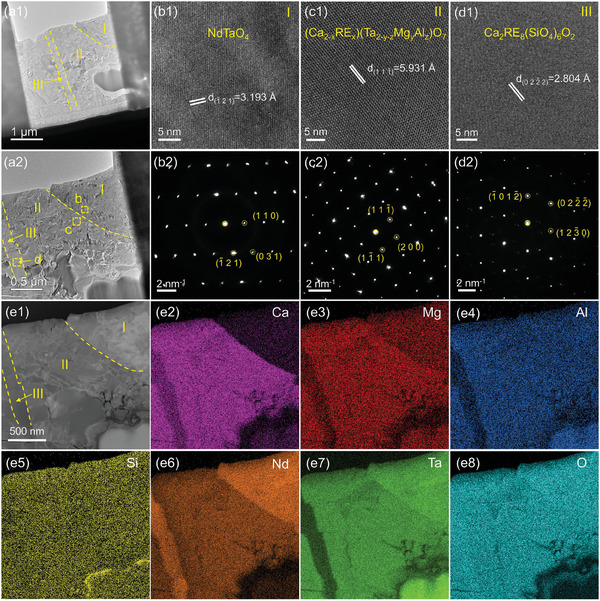
a) TEM images of the reaction front in the NdTaO_4_ after CMAS corrosion at 1300 °C for 50 h; b–d) HRTEM and SAED images of the corresponding regions in (a); e) STEM image and corresponding EDS mapping.

To elucidate the microstructure and composition of the corrosion products, transmission electron microscope (TEM) characterization was conducted. Corrosion products were analyzed from two regions: YTaO_4_, which has smaller RE ionic radius (**Figure** **9**), and NdTaO_4_, which has larger RE ionic radius (**Figure** **10**). The EDS compositional analysis results are summarized in Figures 9, 10, and Table S1 (Supporting Information). Figure 9a distinctly reveals ferroelastic domains in YTaO_4_ (Region I). High‐resolution TEM (HRTEM) and selected area electron diffraction (SAED) images (Figure 9b) confirm the single‐crystalline, ordered monoclinic structure of YTaO_4_, with a (0 4 0) interplanar spacing of 2.784 Å. The compositional analysis reveals that the primary elements are Y, Ta, and O. Figure 9c illustrates the HRTEM and SAED images of the (Ca_2‐x_RE_x_)(Ta_2‐y‐z_Mg_y_Al_z_)O_7_ solid solution, which exhibits a face‐centered cubic structure with a (2 $\bar{2}$ 2) interplanar spacing of 2.807 Å. The EDS mapping and point analysis results indicate the presence of a significant amount of Ca, Y, and Ta, along with a small amount of Mg and Al. Microstructural details of the Ca_2_RE_8_(SiO_4_)_6_O_2_ apatite are presented in Figure 9d, where SAED confirms a hexagonal system with a (0 3$\;\bar{3}\;$ 0) spacing of 2.720 Å, and EDS results reveal the presence of Ca, Y and Si. The microstructure and composition of the corrosion products in the NdTaO_4_ region (Figure 10) closely resemble those in the YTaO_4_ region. SAED patterns confirm the reaction products are face‐centered cubic (Ca_2‐x_RE_x_)(Ta_2‐y‐z_Mg_y_Al_z_)O_7_ and hexagonal Ca_2_RE_8_(SiO_4_)_6_O_2_. Therefore, the corrosion products of different RETaO_4_ after CMAS exposure are consistently (Ca_2‐x_RE_x_)(Ta_2‐y‐z_Mg_y_Al_z_)O_7_ and Ca_2_RE_8_(SiO_4_)_6_O_2_.

**Figure 11:**
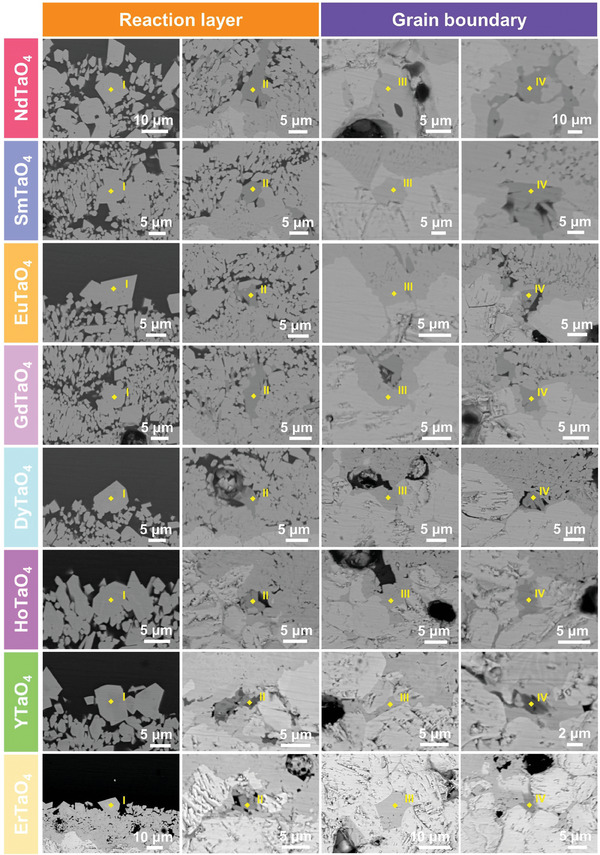
High‐magnification images of corrosion products in the reaction layer and grain boundaries of layered stack RETaO_4_ ceramic after CMAS corrosion at 1300 °C for 25 h.

**Figure 12:**
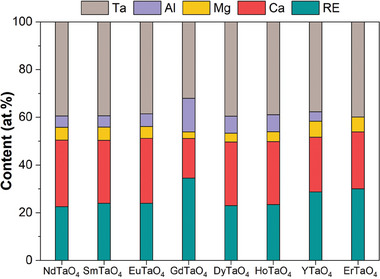
Composition of (Ca_2‐x_RE_x_)(Ta_2‐y‐z_Mg_y_Al_z_)O_7_ in different RETaO_4_ after CMAS corrosion at 1300 °C for 25 h.

Given the limitations of EDS in accurately determining the composition of corrosion products, EPMA was employed for a more precise analysis, utilizing wavelength‐dispersive spectroscopy (WDS) to achieve high‐resolution compositional characterization. **Figure** **11** and **Table** **1** illustrate the EPMA results of the sample after CMAS corrosion for 25 h, with results from 50, 75, and 100 h provided in the supporting information (Figures S1, S3, and S5; Tables S2‐S4, Supporting Information). Point I represents the primary corrosion product within the reaction layer, and its composition in each region is summarized in **Figure** **12**, Figures S2, S4, and S6 (Supporting Information). The content of RE elements in the primary corrosion products generated in different RETaO_4_ regions shows some variation. However, the sum of Ca + RE and Mg + Al+ Ta remains approximately equal to 50%. Based on its phase and microstructure, it can be confirmed that the primary corrosion product is the (Ca_2‐x_RE_x_)(Ta_2‐y‐z_Mg_y_Al_z_)O_7_ solid solution. In the denser reaction layer, a secondary product (Point II) was identified with a Ca:RE ratio of approximately 1:4:3, corresponding to Ca_2_RE_8_(SiO_4_)_6_O_2_ apatite. Point III and Point IV represent two corrosion products in the grain boundaries, respectively. Point III predominates within the grain boundaries, with a composition similar to that of Point I, which is also (Ca_2‐x_RE_x_)(Ta_2‐y‐z_Mg_y_Al_z_)O_7_ solid solution. Point IV shares the same composition as Point II but in lesser amounts, and it is Ca_2_RE_8_(SiO_4_)_6_O_2_ apatite. Thus, the main corrosion products in RETaO_4_ after CMAS exposure at 1300 °C are the (Ca_2‐x_RE_x_)(Ta_2‐y‐z_Mg_y_Al_z_)O_7_ solid solution and minor Ca_2_RE_8_(SiO_4_)_6_O_2_ apatite.

**Table 1:** EPMA analysis of corrosion products of layered stack RETaO_4_ ceramic after CMAS corrosion at 1300 °C for 25 h.

Composition [at.%]															
		Ca	Mg	Al	Si	Ta	O	Nd	Sm	Eu	Gd	Dy	Ho	Y	Er
NdTaO_4_	I	10.22	1.98	1.75	0.00	14.45	63.34	**3.43**	3.64	0.90	0.26	0.00	0.03	0.00	0.00
	II	5.36	0.07	0.00	13.96	0.00	61.70	**10.09**	5.58	1.57	0.67	0.18	0.57	0.18	0.07
	III	8.43	2.76	1.68	0.00	14.18	63.43	**6.16**	3.08	0.20	0.00	0.00	0.05	0.00	0.03
	IV	5.14	0.09	0.00	13.85	0.00	61.72	**15.19**	2.43	1.23	0.00	0.08	0.15	0.08	0.04
SmTaO_4_	I	9.65	2.01	1.76	0.00	14.39	63.42	1.56	**5.00**	1.23	0.53	0.00	0.44	0.00	0.01
	II	5.23	0.12	0.00	13.60	0.00	61.65	3.08	**10.10**	2.40	1.42	0.43	1.40	0.41	0.16
	III	7.86	2.98	1.05	0.00	13.82	63.35	0.47	**8.11**	1.93	0.17	0.00	0.20	0.00	0.06
	IV	5.31	0.06	0.00	13.48	0.00	61.62	3.56	**11.03**	2.04	1.09	0.27	1.11	0.30	0.13
EuTaO_4_	I	10.02	1.80	1.98	0.00	14.13	63.28	0.31	1.18	**4.84**	1.50	0.00	0.96	0.00	0.00
	II	6.03	0.23	0.16	12.15	1.29	61.69	3.37	5.63	**4.51**	2.08	0.31	1.78	0.58	0.19
	III	7.76	2.68	1.55	0.00	13.76	63.42	0.30	0.60	**9.48**	0.24	0.00	0.16	0.00	0.05
	IV	5.12	0.08	0.00	13.64	0.00	61.70	2.36	4.04	**8.97**	1.92	0.00	1.56	0.47	0.14
GdTaO_4_	I	6.08	1.04	5.16	0.00	11.77	63.28	0.00	0.16	3.07	**8.39**	0.00	1.05	0.00	0.00
	II	5.28	0.00	0.00	13.59	0.00	61.66	1.52	2.37	2.30	**10.60**	0.49	1.62	0.51	0.06
	III	8.67	2.18	1.64	0.00	13.90	63.37	0.62	1.12	2.83	**4.71**	0.00	0.88	0.05	0.03
	IV	5.15	0.00	0.00	13.59	0.00	61.70	0.87	1.62	1.64	**10.08**	2.19	2.01	0.89	0.26
DyTaO_4_	I	9.75	1.34	2.59	0.00	14.42	63.54	0.04	0.06	0.11	0.78	**3.91**	1.82	1.17	0.47
	II	5.23	0.00	0.00	14.25	0.00	61.79	0.28	0.67	0.80	3.47	**10.96**	1.63	0.68	0.24
	III	8.87	1.84	2.43	0.00	13.89	63.43	0.13	0.39	0.40	0.89	**4.87**	1.77	0.84	0.25
	IV	5.32	0.02	0.00	13.99	0.00	61.73	0.34	0.82	1.08	2.00	**4.90**	6.86	2.17	0.77
HoTaO_4_	I	9.67	1.52	2.61	0.00	14.22	63.45	0.13	0.51	0.76	1.16	1.37	**2.30**	1.52	0.78
	II	5.47	0.16	0.00	14.23	0.00	61.73	0.07	0.12	0.14	0.34	1.37	**15.03**	1.34	0.00
	III	8.85	1.87	2.28	0.00	13.93	63.44	0.11	0.27	0.36	0.63	0.85	**6.20**	0.91	0.30
	IV	5.40	0.25	0.38	13.06	0.34	61.62	0.03	0.04	0.04	0.22	3.19	**14.78**	0.65	0.00
YTaO_4_	I	8.39	2.44	1.46	0.00	13.81	63.36	0.01	0.01	0.06	0.09	0.15	1.75	**8.12**	0.35
	II	5.67	0.35	0.00	11.70	0.93	61.50	0.03	0.06	0.07	0.11	0.10	0.18	**16.62**	2.68
	III	6.75	1.47	3.55	0.00	12.92	63.53	0.00	0.00	0.02	0.04	0.07	0.48	**8.85**	2.32
	IV	5.23	0.33	0.14	11.86	1.60	61.90	0.05	0.12	0.12	0.22	0.28	1.16	**15.55**	1.44
ErTaO_4_	I	8.69	2.28	0.00	0.00	14.51	63.60	0.03	0.01	0.00	0.08	0.03	0.15	1.82	**8.80**
	II	5.14	0.21	0.00	13.92	0.00	61.72	0.10	0.21	0.18	0.42	0.50	0.80	4.91	**11.89**
	III	0.62	1.82	6.57	0.00	10.88	63.87	0.00	0.00	0.00	0.00	0.03	0.01	3.53	**12.67**
	IV	5.01	0.27	0.00	13.72	0.00	61.71	0.00	0.01	0.00	0.05	0.01	0.26	4.51	**14.45**

## 3 Discussion

Through precise analysis of the microstructure (TEM) and composition (EPMA) of corrosion products, it is evident that the following reactions occurred in the RETaO_4_ ceramics during the CMAS attack:

(1) $$\begin{array}{ccc} & & \left(2-x\right)\mathrm{CaO}+\mathrm{yMgO}+z/2\;Al_{2}O_{3\;}\;+\;\mathrm{xRETa}O_{4}\;+\;\left(2-x-y-z\right)/\\ & & 2\;Ta_{2}O_{5}\to \left(Ca_{2-x}RE_{x}\right)\left(Ta_{2-y-z}Mg_{y}Al_{z}\right)O_{7} \end{array}$$

(2) $$2\mathrm{CaO}+4RE_{2}O_{3}+6\mathrm{Si}O_{2}\to Ca_{2}RE_{8}{\left(\mathrm{Si}O_{4}\right)}_{6}O_{2}$$

**Figure 13:**
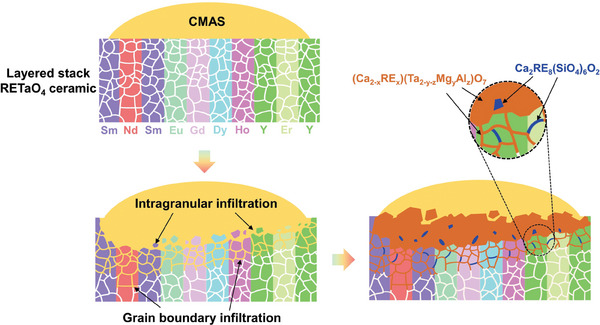
Schematic diagram of CMAS corrosion behavior of the layered stack RETaO_4_ ceramics at 1300 °C.

Based on EPMA analysis (Table 1), the content of Ta in the (Ca_2‐x_RE_x_)(Ta_2‐y‐z_Mg_y_Al_z_)O_7_ solid solution exceeds that of RE, suggesting an excess of Ta_2_O_5_ in forming the solid solution. **Figure** **13** presents a schematic diagram of the CMAS corrosion behavior in layered stack RETaO_4_ ceramics. At high temperatures, CMAS melts and spreads across the RETaO_4_ ceramic surface, dissolving the material and infiltrating its grain boundaries. As elements in the liquid CMAS reach saturation, a dense reaction layer forms at the interface between CMAS and the RETaO_4_ substrate. Corrosion products also form along the grain boundaries, primarily as (Ca_2‐x_RE_x_)(Ta_2‐y‐z_Mg_y_Al_z_)O_7_ solid solutions, along with minor Ca_2_RE_8_(SiO_4_)_6_O_2_ apatite. Additionally, the infiltration depth of CMAS decreases as the RE ionic radius decreases.

**Figure 14:**
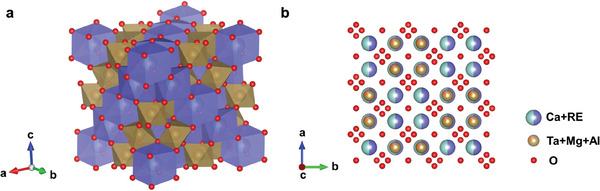
Schematic diagram of the crystal structure of (Ca_0.5_RE_0.5_)_2_(Ta_0.75_Mg_0.125_Al_0.125_)_2_O_7_ solid solution. a) Polyhedral model, b) View along the c‐axis.

**Figure 15:**
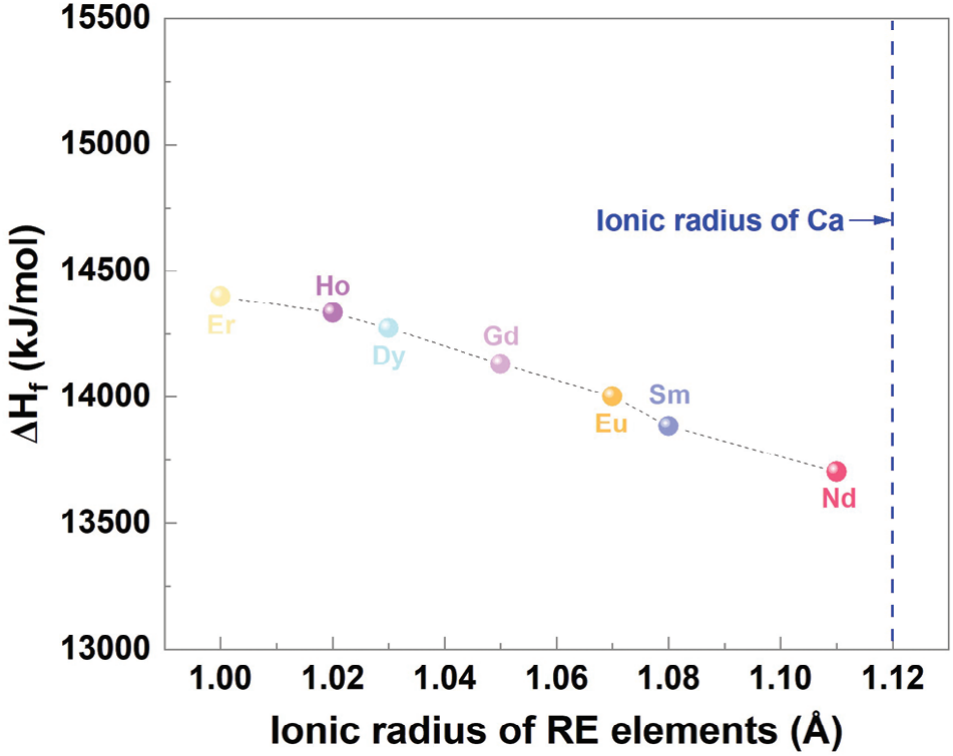
The relationship between the formation enthalpy of (Ca_0.5_RE_0.5_)_2_(Ta_0.75_Mg_0.125_Al_0.125_)_2_O_7_ and the RE ionic radius.

To further investigate the effect of RE species on the CMAS corrosion resistance of RETaO_4_ ceramics, the formation enthalpy of the primary corrosion products (Ca_2‐x_RE_x_)(Ta_2‐y‐z_Mg_y_Al_z_)O_7_ was calculated. As shown in Table 1, the atomic ratio of Ca in this solid solution ranges from 0.65 to 1.11, while RE varies between 0.89 and 1.35. Ta has an atomic ratio of about 1.5, with Mg and Al each around 0.25. For calculation purposes, the composition of the (Ca_2‐x_RE_x_)(Ta_2‐y‐z_Mg_y_Al_z_)O_7_ solid solution is defined as (Ca_0.5_RE_0.5_)_2_(Ta_0.75_Mg_0.125_Al_0.125_)_2_O_7_. **Figure** **14** exhibits the crystal structure of (Ca_0.5_RE_0.5_)_2_(Ta_0.75_Mg_0.125_Al_0.125_)_2_O_7_, with a space group of *Fd* $\bar{3}$ *m*. In this structure, Ca and RE are eight‐coordinated, while Ta, Mg, and Al are six‐coordinated. The calculated formation enthalpies for the (Ca_0.5_RE_0.5_)_2_(Ta_0.75_Mg_0.125_Al_0.125_)_2_O_7_ solid solutions corresponding to each RE element are shown in **Figure** **15**. The formation enthalpy of (Ca_0.5_RE_0.5_)_2_(Ta_0.75_Mg_0.125_Al_0.125_)_2_O_7_ decreases gradually with increasing RE ionic radius, indicating that larger RE ionic radius result in more exothermic formation enthalpies and easier formation of (Ca_0.5_RE_0.5_)_2_(Ta_0.75_Mg_0.125_Al_0.125_)_2_O_7_. Additionally, when the ionic radius of RE approaches that of Ca, it becomes easier for RE to substitute for Ca in the solid solution. For another corrosion product, Ca_2_RE_8_(SiO_4_)_6_O_2_ apatite, Costa et al. also demonstrated that its formation enthalpy is negatively correlated with the RE ionic radius.^[^ ^43^ ^]^ As a result, a larger RE ionic radius leads to easier formation of corrosion products in RETaO_4_ during CMAS corrosion processes, thereby causing more severe degradation.

**Figure 16:**
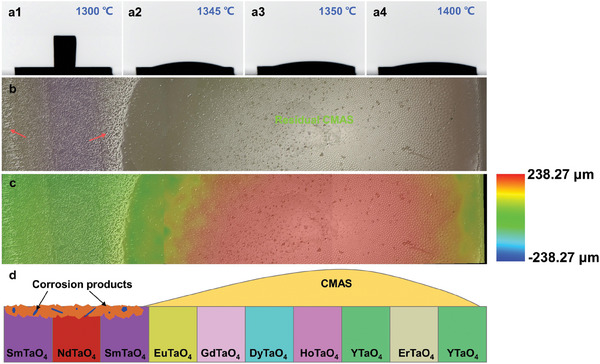
a) Wetting behavior of CMAS molten salt on layered stack RETaO_4_ ceramic at high temperatures; b) Optical micrograph of the sample surface after conducting high‐temperature CMAS wetting behavior experiments; c) Ultra‐depth‐of‐field micrograph of b; d) Schematic diagram of CMAS wetting behavior on layered stack RETaO_4_ ceramic at high temperatures.

At high temperatures, CMAS melts and wets the surface of the sample. With the increase of time, it penetrates and reacts with the substrate. The wetting behavior between liquid CMAS and the substrate reflects the CMAS corrosion resistance, characterized by the contact angle between the liquid CMAS and the substrate. A larger contact angle indicates poor wetting and, consequently, better corrosion resistance. **Figure** **16**a shows the wetting behavior of CMAS molten salt on layered stack RETaO_4_ ceramic at high temperatures. At 1345 °C, CMAS uniformly spreads on the sample surface. By 1350 °C, the molten CMAS preferentially spreads toward the right end (where YTaO_4_ is located), while no CMAS is present at the left end (where SmTaO_4_ is located), even as the temperature reaches 1400 °C. The sample characterized for high‐temperature CMAS wetting behavior was observed using an ultra‐depth‐of‐field microscope (Figure 16b,c). On the right side of the sample, a significant amount of residual CMAS was observed, whereas the left side displayed granular and rod‐shaped corrosion products (indicated by the red arrows), indicating complete reaction of CMAS with the substrate. Figure 16c shows the surface height variation across the sample, where the contact angle is smaller on the left side but steeper on the right, indicating greater resistance to CMAS corrosion on the right side. Figure 16d depicts a schematic diagram of the wetting behavior of CMAS molten salt on layered stack RETaO_4_ ceramic at high temperatures. On the left side, CMAS spread rapidly and reacted completely, resulting in a very small contact angle. This indicated excellent wetting between CMAS and the substrate, suggesting that RETaO_4_ with a larger RE ionic radius (such as NdTaO_4_ and SmTaO_4_) have poorer resistance to CMAS attack. On the right side, there was more residual CMAS, and the contact angle was larger. This indicated that RETaO_4_ with a smaller RE ionic radius (such as ErTaO_4_ and YTaO_4_) have poorer wetting with CMAS, and exhibited chemical inertness, demonstrating excellent resistance to CMAS corrosion.

## 4 Conclusion 

This study employ a high‐throughput approach to systematically compare the CMAS corrosion behavior of RETaO_4_ (RE = Nd, Sm, Eu, Gd, Dy, Ho, Y, and Er) at 1300 °C. Corrosion products are primarily composed of (Ca_2‐x_RE_x_)(Ta_2‐y‐z_Mg_y_Al_z_)O_7_ solid solutions, with minor amounts of Ca_2_RE_8_(SiO_4_)_6_O_2_ apatite. The depth of CMAS infiltration increases with the RE ionic radius, as larger RE ions lead to more exothermic enthalpy and facilitate easier formation of corrosion products. Wetting behavior experiments further confirmed that RETaO_4_ ceramics with smaller RE ionic radius experience better CMAS corrosion. These findings provide valuable insights for designing RETaO_4_‐based TBCs with enhanced CMAS corrosion resistance.

## 5 Experimental Procedure and Computation Methods

### Preparation Process

**Figure 17:**
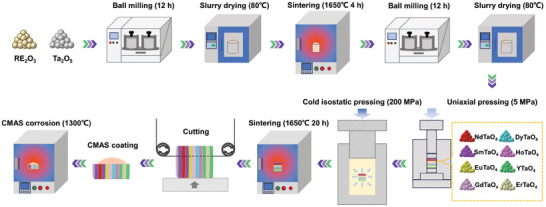
Schematic diagram of the preparation of layered stack RETaO_4_ ceramics.

The preparation process of layered stack RETaO_4_ ceramics is shown in **Figure** **17**. The RE_2_O_3_ (RE = Nd, Sm, Eu, Gd, Dy, Ho, Y, and Er) and Ta_2_O_5_ powder was weighed according to the molar ratio of 1:1 and ball milled with ethanol as the medium for 12 h at a rotation speed of 300 r min^−1^. The ball‐milled slurry was dried in an oven at 80 °C for 12 h, passed through a 60‐mesh sieve, and then sintered in a muffle furnace at 1650 °C for 4 h to obtain pure phase RETaO_4_ powder. The RETaO_4_ powder was ball‐milled again for 12 h. The ball‐milled slurry was dried in an oven at 80 °C for 12 h and passed through a 60‐mesh sieve to obtain fine RETaO_4_ powder. Add the RETaO_4_ powder to the mold in order of SmTaO_4_‐NdTaO_4_‐SmTaO_4_‐EuTaO_4_‐GdTaO_4_‐DyTaO_4_‐HoTaO_4_‐YTaO_4_‐ErTaO_4_‐YTaO_4_. The thickness of each layer is controlled to be roughly equal according to the density and weight of each powder. Each time a powder is added, press it once at 1 MPa. After all the powder is added, press it at 5 MPa for 5 min. An extra layer of SmTaO_4_ and YTaO_4_ at both ends will not be covered by CMAS molten salt, serving as a benchmark for measuring CMAS infiltration depth, while ensuring that each layer in the middle is completely covered by CMAS molten salt. The prefabricated body is isostatically cold pressed at 200 MPa to obtain the green body. The green body was sintered in a muffle furnace at 1650 °C for 20 h to obtain layered stack RETaO_4_ ceramics.

### CMAS Reaction

The composition of CMAS molten salt used in this work was 33CaO‐9MgO‐13AlO_1.5_‐45SiO_2_. CaO, MgO, Al_2_O_3_, and SiO_2_ powder were weighed according to the composition of CMAS and then ball‐milled at 300 r min^−1^ for 12 h. The ball‐milled slurry was dried in an oven at 80 °C for 12 h, passed through a 60‐mesh sieve, and then sintered in a muffle furnace at 1200 °C for 24 h to synthesize CMAS molten salt. The CMAS molten salt was ball‐milled, dried, and passed through a 60‐mesh sieve again to obtain fine CMAS powder. As shown in Figure 14, the layered stack RETaO_4_ ceramics were cut along the radial direction. The CMAS powder was mixed with ethanol and coated on the cut surface of the sample, the CMAS loading was 30 mg cm^−2^. The CMAS‐coated samples were heated in a muffle furnace at 1300 °C for 25, 50, 75, and 100 h, respectively.

### Characterization

The phase compositions of the samples were analyzed by X‐ray diffractometer (XRD, PANalytical, Empyrean, Netherlands). Scanning electron microscope (SEM, Hitachi, SU5000, Japan) and energy dispersive spectrometer (EDS, Bruker, Xflash6‐30, Germany) were used to characterize the microstructure and elemental distribution. Transmission electron microscope (TEM, JEOL, JEM‐3200FS, Japan) was employed to characterize the microstructure of the corrosion reaction front. Electron probe microanalyzer (EPMA, JEOL, JXA‐8530F Plus, Japan) was used to specify the exact composition of the corrosion products. The wetting behavior of CMAS molten salts with samples at high temperatures was evaluated using a self‐built high‐temperature contact angle measurement system. The surface morphology of the sample after the high‐temperature CMAS wetting behavior experiment was observed using an ultra‐depth‐of‐field microscope (CDLY‐TECH, LY‐WN‐YH400, China).

### Computation Methods

The first‐principles calculation of the formation enthalpy of corrosion products is based on density functional theory (DFT).^[^ ^44^ ^]^ Vienna Ab‐Initio Simulation Package (VASP) was chosen for computation, employing the generalized gradient approximation (GGA) functional and projected‐augmented‐waves (PAW) method to handle electron‐ion interactions.^[^ ^45^, ^46^ ^]^ The plane wave energy cutoff was set to 520 eV, and a spacing of 0.3 Å^−1^ with the Monkhorst‐Pack method was used for Brillouin zone sampling.^[^ ^47^ ^]^ Convergence for the formation enthalpy calculations was achieved with a total energy convergence criterion of 1 × 10^−7^ eV.

## Conflict of Interest

The authors declare no conflict of interest.

## Supporting information



Supporting Information

## Data Availability

The data that support the findings of this study are available from the corresponding author upon reasonable request.
