# Supplementary material for: High Throughput Screening of CMAS Corrosion‐Resistant RETaO4 Based on Lamination Method
Source: Adv Sci (Weinh). 2025 Feb 17;12(14):2412717. doi: 10.1002/advs.202412717 (PMC11984857; doi:10.1002/advs.202412717)
Supplement: Supplementary file 1 — Supporting Information [file ADVS-12-2412717-s001.docx]

**High Throughput Screening of CMAS Corrosion-resistant RETaO_4_ Based on Lamination Method**

*Zhilin Tian*^1^, Zhilin Chen^1^, Shuping Wen, Wenxia Zhao, Liya Zheng, Bin Li**

Zhilin Tian, Zhilin Chen, Shuping Wen, Liya Zheng, Bin Li

School of Materials, Shenzhen Campus of Sun Yat-sen University, Shenzhen, 518107, China

E-mail: tianzhlin@mail.sysu.edu.cn, libin75@mail.sysu.edu.cn

Zhilin Tian

Guangdong-Hong Kong Joint Laboratory of Modern Surface Engineering Technology, Guangdong Provincial Key Laboratory of Modern Surface Engineering Technology, Institute of New Materials, Guangdong Academy of Sciences, Guangzhou, 510650, China

Wenxia Zhao

Instrumental analysis and research center, Sun Yat-sen University, Guangzhou, 510275, China

| Composition (at.%) | | | | | | | | |
| --- | --- | --- | --- | --- | --- | --- | --- | --- |
|  |  | O | Mg | Al | Si | Ca | RE | Ta |
| YTaO_4_ | I | 72.5 | 0.0 | 0.0 | 4.9 | 0.0 | 9.0 | 13.5 |
|  | II | 71.8 | 1.0 | 3.0 | 3.7 | 4.3 | 6.0 | 10.2 |
|  | III | 72.0 | 0.3 | 0.0 | 12.3 | 3.5 | 11.4 | 0.5 |
| NdTaO_4_ | I | 68.8 | 0.1 | 0.2 | 3.9 | 0.0 | 13.5 | 13.5 |
|  | II | 70.1 | 1.5 | 1.4 | 3.8 | 5.9 | 5.5 | 11.9 |
|  | III | 75.0 | 0.0 | 0.0 | 10.7 | 3.2 | 10.3 | 0.7 |

**Table S1** EDS point analysis of the reaction front in YTaO_4_ and NdTaO_4_ in Figure 9 and 10.


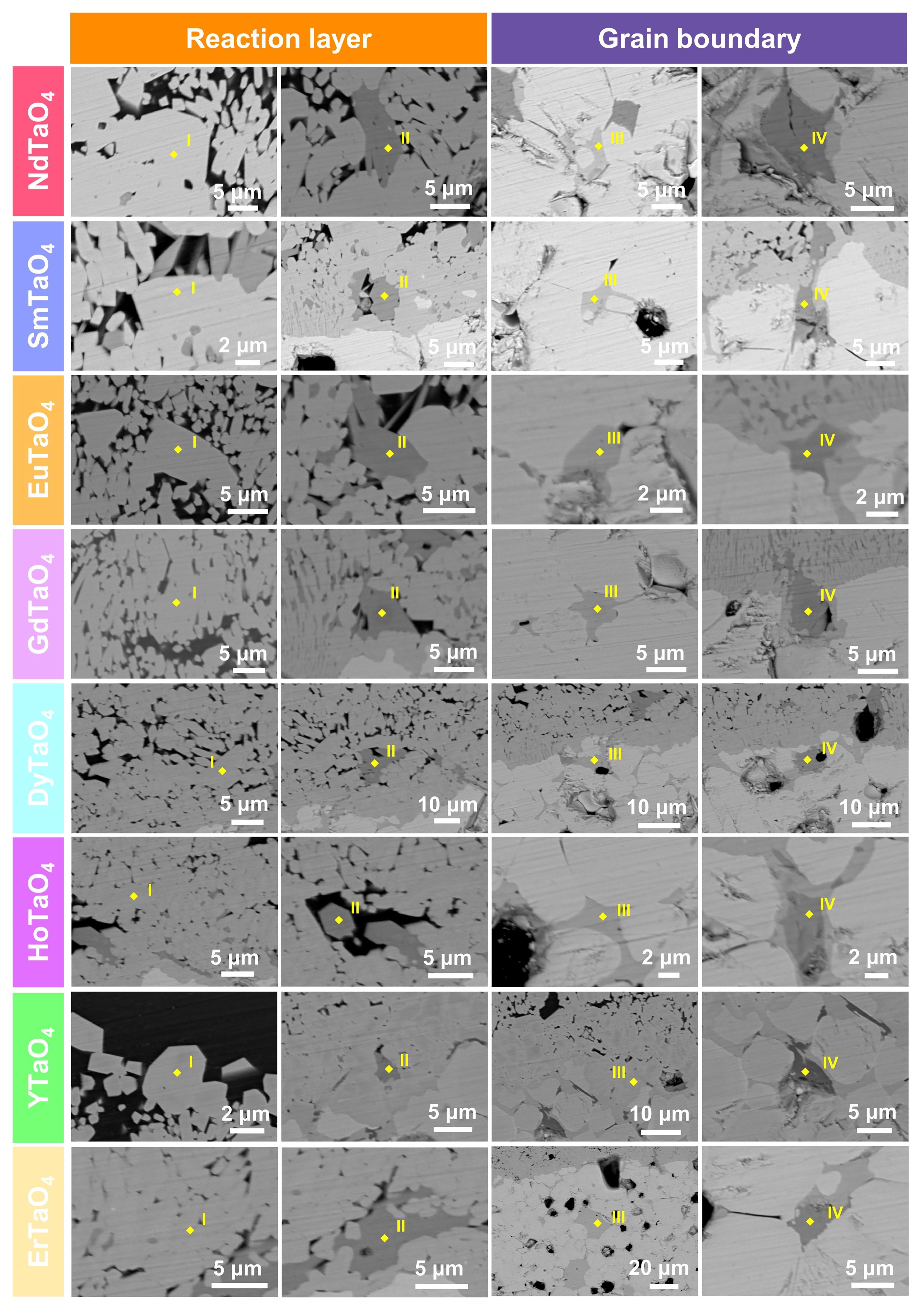


**Figure S1.** High-magnification images of corrosion products at the reaction layer and grain boundaries of layered stack RETaO_4_ ceramic after CMAS corrosion at 1300^o^C for 50 h.


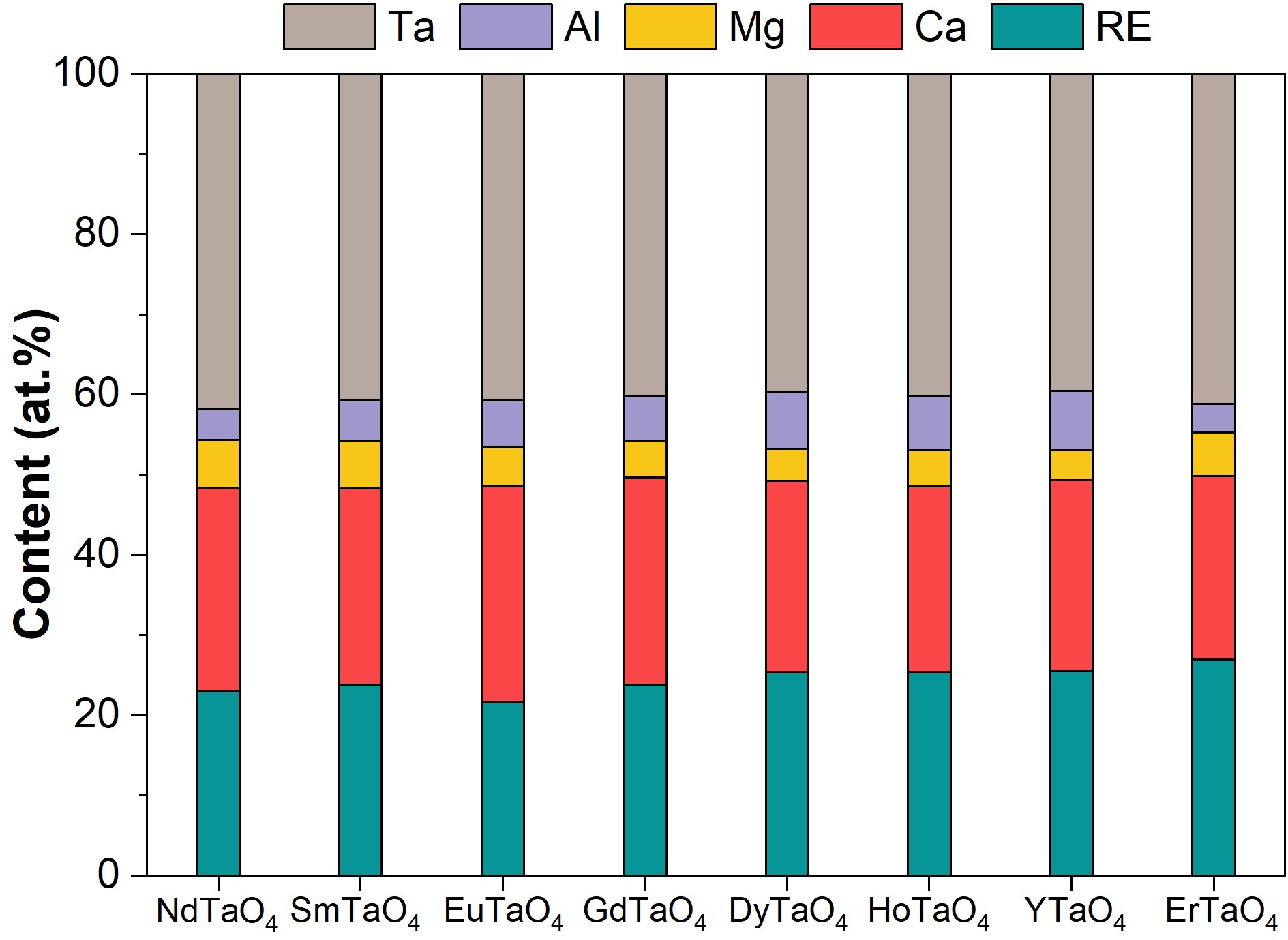


**Figure S2.** Composition of (Ca_2-x_RE_x_)(Ta_2-y-z_Mg_y_Al_z_)O_7_ in different RETaO_4_ after CMAS corrosion at 1300℃ for 50 h.


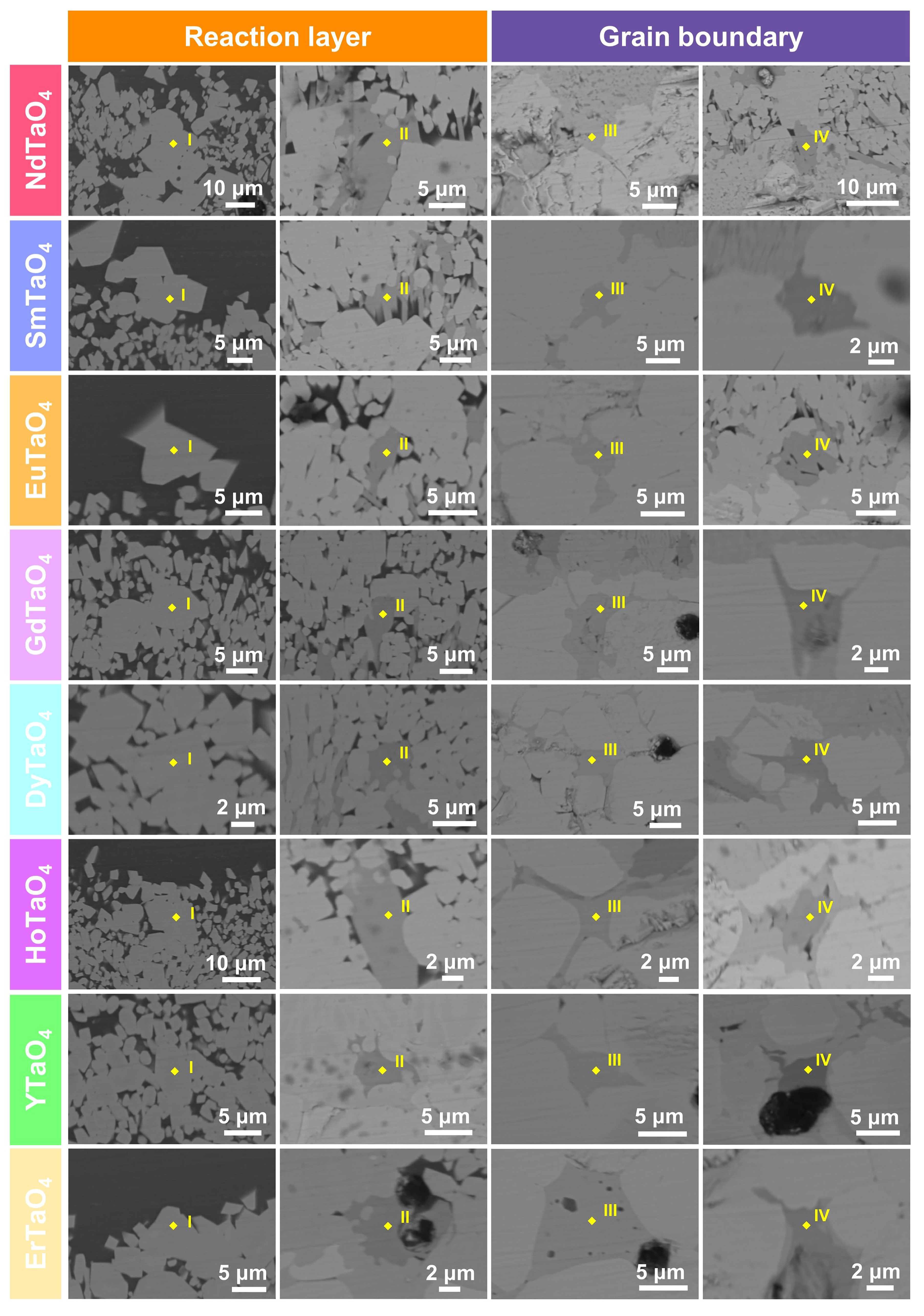


**Figure S3.** High-magnification images of corrosion products at the reaction layer and grain boundaries of layered stack RETaO_4_ ceramic after CMAS corrosion at 1300^o^C for 75 h.


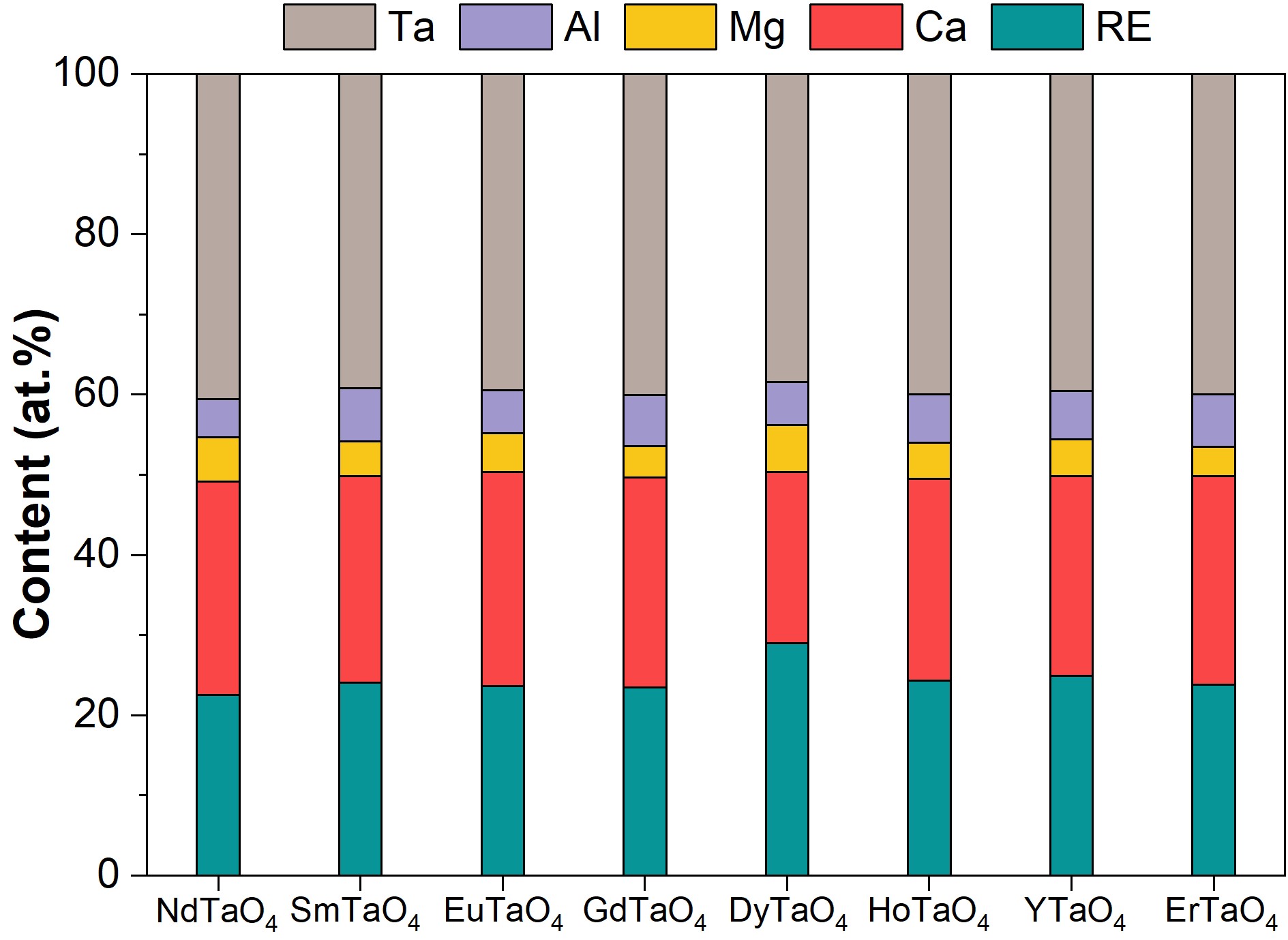


**Figure S4.** Composition of (Ca_2-x_RE_x_)(Ta_2-y-z_Mg_y_Al_z_)O_7_ in different RETaO_4_ after CMAS corrosion at 1300℃ for 75 h.


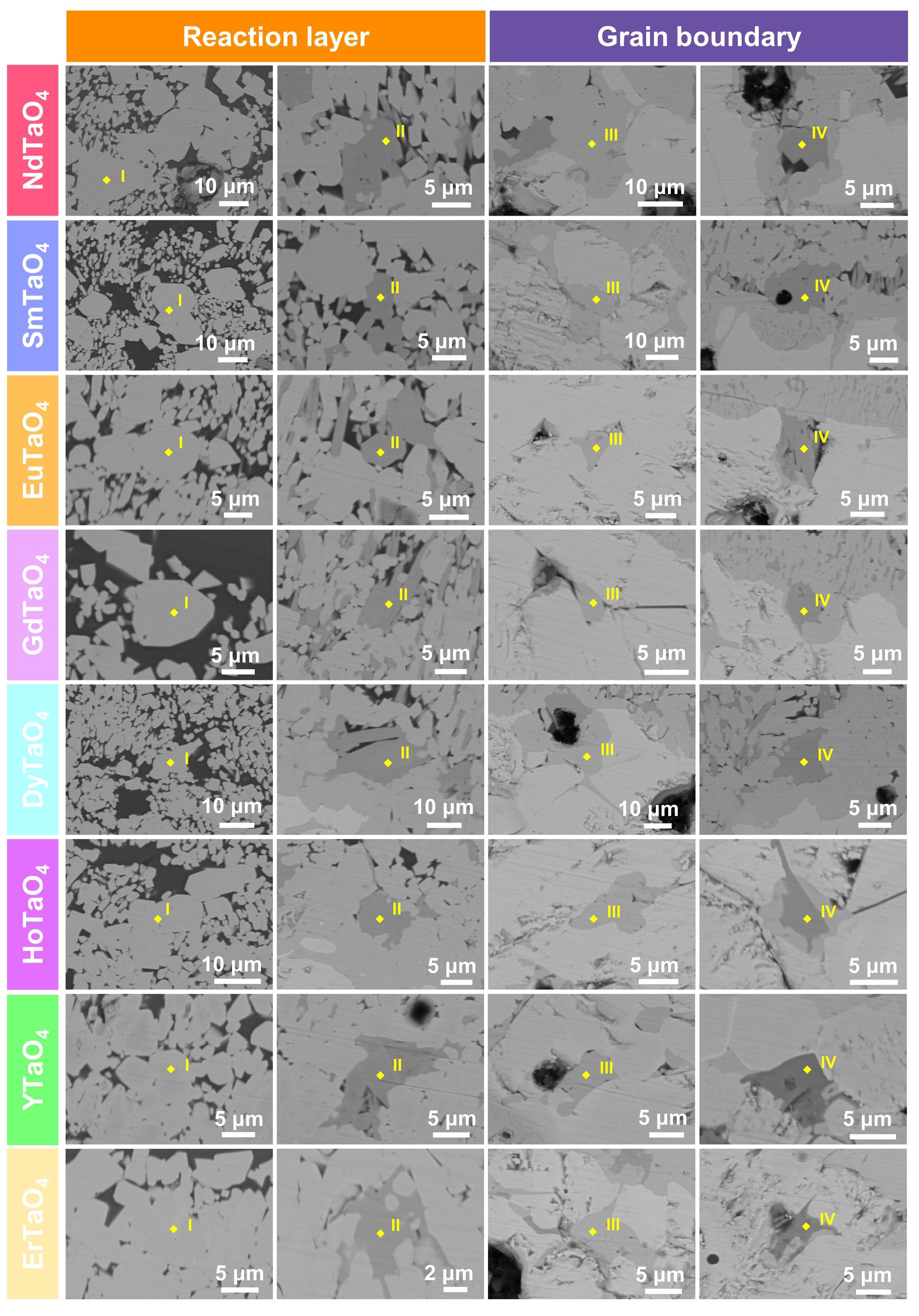


**Figure S5.** High-magnification images of corrosion products at the reaction layer and grain boundaries of layered stack RETaO_4_ ceramic after CMAS corrosion at 1300^o^C for 100 h.


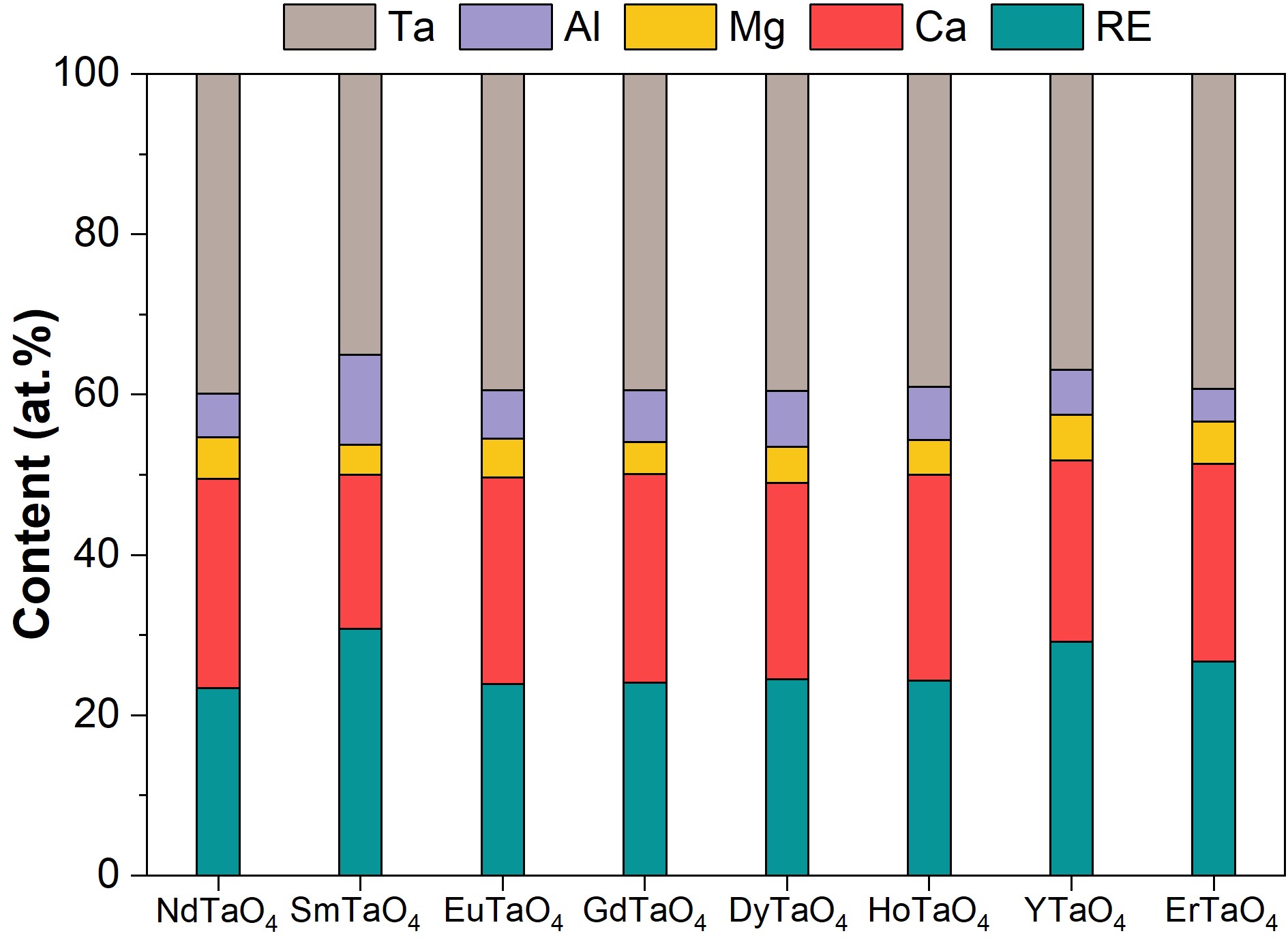


**Figure S6.** Composition of (Ca_2-x_RE_x_)(Ta_2-y-z_Mg_y_Al_z_)O_7_ in different RETaO_4_ after CMAS corrosion at 1300℃ for 100 h.

**Table S2.** EPMA analysis of corrosion products of layered stack RETaO_4_ ceramic after CMAS corrosion at 1300^o^C for 50 h

| Composition (at.%) | | | | | | | | | | | | | | | |
| --- | --- | --- | --- | --- | --- | --- | --- | --- | --- | --- | --- | --- | --- | --- | --- |
|  |  | Ca | Mg | Al | Si | Ta | O | Nd | Sm | Eu | Gd | Dy | Ho | Y | Er |
| NdTaO_4_ | I | 8.95 | 2.12 | 1.34 | 0.74 | 14.82 | 63.88 | **5.40** | 1.53 | 0.55 | 0.22 | 0.21 | 0.20 | 0.02 | 0.02 |
|  | II | 5.34 | 0.02 | 0.00 | 14.57 | 0.00 | 61.86 | **6.09** | 5.90 | 2.72 | 1.55 | 0.37 | 0.94 | 0.46 | 0.18 |
|  | III | 7.37 | 2.34 | 1.80 | 0.48 | 14.73 | 64.03 | **8.49** | 0.48 | 0.15 | 0.13 | 0.00 | 0.00 | 0.00 | 0.00 |
|  | IV | 4.83 | 0.07 | 0.01 | 14.55 | 0.00 | 61.93 | **15.87** | 1.25 | 1.28 | 0.00 | 0.03 | 0.13 | 0.05 | 0.00 |
| SmTaO_4_ | I | 8.75 | 2.13 | 1.81 | 0.51 | 14.55 | 63.75 | 1.03 | **5.58** | 0.73 | 0.42 | 0.30 | 0.38 | 0.06 | 0.00 |
|  | II | 5.00 | 0.08 | 0.00 | 14.50 | 0.14 | 61.93 | 2.10 | **13.22** | 0.83 | 0.91 | 0.43 | 0.63 | 0.22 | 0.01 |
|  | III | 5.40 | 1.17 | 4.66 | 0.44 | 12.34 | 63.70 | 1.44 | **10.59** | 0.24 | 0.02 | 0.00 | 0.00 | 0.00 | 0.00 |
|  | IV | 4.77 | 0.13 | 0.00 | 14.34 | 0.03 | 61.89 | 2.65 | **15.22** | 0.57 | 0.28 | 0.06 | 0.03 | 0.03 | 0.00 |
| EuTaO_4_ | I | 9.80 | 1.78 | 2.10 | 0.00 | 14.83 | 63.61 | 0.90 | 1.53 | **3.20** | 1.01 | 0.00 | 0.66 | 0.39 | 0.19 |
|  | II | 5.05 | 0.00 | 0.00 | 14.44 | 0.00 | 61.87 | 1.70 | 2.78 | **10.24** | 2.33 | 0.51 | 0.62 | 0.33 | 0.13 |
|  | III | 5.37 | 0.86 | 4.88 | 0.00 | 12.16 | 63.62 | 0.08 | 0.47 | **10.88** | 1.38 | 0.00 | 0.21 | 0.03 | 0.06 |
|  | IV | 4.75 | 0.21 | 0.10 | 13.32 | 0.65 | 61.93 | 0.22 | 1.05 | **15.74** | 1.18 | 0.64 | 0.11 | 0.07 | 0.03 |
| GdTaO_4_ | I | 9.39 | 1.69 | 1.99 | 0.00 | 14.64 | 63.65 | 1.11 | 2.11 | 1.55 | **2.23** | 0.38 | 0.84 | 0.26 | 0.16 |
|  | II | 5.20 | 0.00 | 0.00 | 14.30 | 0.00 | 61.82 | 1.75 | 3.17 | 2.99 | **7.76** | 0.63 | 1.58 | 0.63 | 0.17 |
|  | III | 4.92 | 0.75 | 5.56 | 0.00 | 11.87 | 63.61 | 0.00 | 0.06 | 0.60 | **10.31** | 1.06 | 1.26 | 0.00 | 0.00 |
|  | IV | 5.07 | 0.00 | 0.00 | 14.30 | 0.00 | 61.86 | 0.92 | 1.72 | 1.59 | **9.40** | 2.01 | 1.96 | 0.87 | 0.30 |
| DyTaO_4_ | I | 8.53 | 1.43 | 2.54 | 0.51 | 14.17 | 63.78 | 0.24 | 0.56 | 0.88 | 1.20 | **3.16** | 2.11 | 0.89 | 0.00 |
|  | II | 5.09 | 0.00 | 0.00 | 14.50 | 0.05 | 61.91 | 0.40 | 0.86 | 1.33 | 2.37 | **7.83** | 3.88 | 1.78 | 0.00 |
|  | III | 7.82 | 1.64 | 2.29 | 1.32 | 13.30 | 63.69 | 0.19 | 0.34 | 0.45 | 0.95 | **6.07** | 1.49 | 0.45 | 0.00 |
|  | IV | 4.78 | 0.10 | 0.07 | 14.43 | 0.06 | 61.94 | 0.04 | 0.07 | 0.07 | 0.80 | **14.72** | 2.74 | 0.18 | 0.00 |
| HoTaO_4_ | I | 8.29 | 1.63 | 2.42 | 0.49 | 14.32 | 63.82 | 0.08 | 0.18 | 0.30 | 0.52 | 2.06 | **5.24** | 0.65 | 0.00 |
|  | II | 5.05 | 0.01 | 0.00 | 14.23 | 0.27 | 61.94 | 0.49 | 1.07 | 1.54 | 2.69 | 3.63 | **6.04** | 2.78 | 0.26 |
|  | III | 4.07 | 0.74 | 6.27 | 0.49 | 11.76 | 63.82 | 0.00 | 0.01 | 0.02 | 0.16 | 2.33 | **10.04** | 0.29 | 0.00 |
|  | IV | 4.90 | 0.11 | 0.00 | 14.28 | 0.06 | 61.88 | 0.05 | 0.04 | 0.04 | 0.24 | 3.12 | **14.73** | 0.55 | 0.00 |
| YTaO_4_ | I | 8.23 | 1.28 | 2.53 | 1.71 | 13.61 | 63.87 | 0.01 | 0.03 | 0.05 | 0.09 | 0.43 | 1.10 | **4.69** | 2.37 |
|  | II | 5.16 | 0.02 | 0.00 | 14.56 | 0.00 | 61.88 | 0.40 | 0.95 | 1.42 | 2.34 | 2.89 | 4.56 | **5.20** | 0.62 |
|  | III | 8.34 | 1.74 | 2.30 | 0.51 | 14.37 | 63.81 | 0.17 | 0.44 | 0.63 | 0.88 | 1.18 | 1.84 | **3.65** | 0.14 |
|  | IV | 4.92 | 0.23 | 0.00 | 14.19 | 0.73 | 62.10 | 0.05 | 0.05 | 0.10 | 0.09 | 0.20 | 3.32 | **13.63** | 0.39 |
| ErTaO_4_ | I | 8.13 | 1.94 | 1.25 | 0.49 | 14.66 | 63.94 | 0.08 | 0.19 | 0.32 | 0.46 | 0.73 | 0.96 | 1.46 | **5.39** |
|  | II | 5.24 | 0.21 | 0.00 | 13.48 | 1.25 | 62.09 | 0.31 | 0.62 | 0.95 | 1.58 | 2.13 | 3.21 | 3.25 | **5.68** |
|  | III | 0.39 | 1.61 | 6.10 | 1.24 | 10.91 | 64.22 | 0.00 | 0.00 | 0.00 | 0.00 | 0.03 | 0.00 | 2.34 | **13.16** |
|  | IV | 4.81 | 0.19 | 0.00 | 14.67 | 0.00 | 61.93 | 0.08 | 0.11 | 0.11 | 0.19 | 0.24 | 0.37 | 2.39 | **14.91** |

| Composition (at.%) | | | | | | | | | | | | | | | |
| --- | --- | --- | --- | --- | --- | --- | --- | --- | --- | --- | --- | --- | --- | --- | --- |
|  |  | Ca | Mg | Al | Si | Ta | O | Nd | Sm | Eu | Gd | Dy | Ho | Y | Er |
| NdTaO_4_ | I | 9.71 | 2.03 | 1.73 | 0.00 | 14.78 | 63.55 | **2.73** | 2.37 | 1.03 | 0.72 | 0.36 | 0.55 | 0.24 | 0.20 |
|  | II | 5.24 | 0.00 | 0.00 | 14.01 | 0.00 | 61.76 | **4.46** | 4.69 | 2.31 | 2.34 | 1.46 | 1.61 | 1.51 | 0.61 |
|  | III | 8.18 | 2.41 | 1.44 | 0.00 | 14.43 | 63.66 | **7.35** | 1.24 | 0.63 | 0.00 | 0.16 | 0.29 | 0.11 | 0.10 |
|  | IV | 5.17 | 0.04 | 0.00 | 14.49 | 0.00 | 61.85 | **5.33** | 4.34 | 2.12 | 1.97 | 1.30 | 1.47 | 1.39 | 0.53 |
| SmTaO_4_ | I | 9.39 | 1.60 | 2.43 | 0.00 | 14.29 | 63.52 | 1.04 | **2.88** | 1.53 | 1.10 | 0.72 | 0.79 | 0.41 | 0.30 |
|  | II | 5.30 | 0.10 | 0.00 | 14.39 | 0.00 | 61.80 | 3.02 | **6.81** | 2.21 | 2.03 | 1.17 | 1.43 | 1.17 | 0.57 |
|  | III | 5.32 | 1.28 | 4.84 | 0.00 | 12.35 | 63.63 | 1.07 | **10.78** | 0.64 | 0.00 | 0.00 | 0.06 | 0.00 | 0.03 |
|  | IV | 4.70 | 0.13 | 0.00 | 13.75 | 0.00 | 61.77 | 1.08 | **16.51** | 2.03 | 0.00 | 0.00 | 0.03 | 0.00 | 0.00 |
| EuTaO_4_ | I | 9.76 | 1.78 | 1.97 | 0.00 | 14.41 | 63.45 | 1.09 | 2.06 | **2.02** | 1.08 | 0.64 | 0.86 | 0.50 | 0.38 |
|  | II | 5.22 | 0.00 | 0.00 | 13.94 | 0.00 | 61.75 | 1.82 | 3.17 | **8.99** | 1.81 | 0.00 | 1.89 | 0.95 | 0.46 |
|  | III | 4.76 | 1.05 | 5.39 | 0.00 | 11.86 | 63.57 | 0.11 | 1.53 | **11.12** | 0.49 | 0.00 | 0.11 | 0.00 | 0.01 |
|  | IV | 4.85 | 0.10 | 0.00 | 13.76 | 0.00 | 61.76 | 0.88 | 2.04 | **13.51** | 1.44 | 0.00 | 1.06 | 0.43 | 0.17 |
| GdTaO_4_ | I | 9.51 | 1.44 | 2.32 | 0.00 | 14.56 | 63.64 | 0.39 | 0.81 | 0.61 | **1.88** | 1.61 | 1.29 | 1.23 | 0.71 |
|  | II | 5.03 | 0.00 | 0.00 | 13.88 | 0.00 | 61.77 | 0.96 | 1.38 | 1.22 | **9.51** | 3.35 | 1.85 | 0.80 | 0.25 |
|  | III | 8.52 | 1.79 | 2.16 | 0.00 | 13.96 | 63.51 | 0.27 | 0.40 | 0.32 | **4.19** | 3.90 | 0.75 | 0.18 | 0.05 |
|  | IV | 4.78 | 0.00 | 0.00 | 12.88 | 0.00 | 61.61 | 0.16 | 0.30 | 1.95 | **16.06** | 0.40 | 1.78 | 0.08 | 0.00 |
| DyTaO_4_ | I | 7.79 | 2.13 | 1.96 | 0.00 | 13.99 | 63.59 | 0.21 | 0.38 | 0.30 | 0.80 | **7.62** | 0.76 | 0.30 | 0.17 |
|  | II | 5.23 | 0.01 | 0.00 | 14.33 | 0.00 | 61.81 | 1.54 | 2.41 | 1.79 | 2.67 | **5.18** | 2.25 | 1.95 | 0.83 |
|  | III | 4.62 | 0.80 | 6.29 | 0.00 | 11.87 | 63.67 | 0.03 | 0.00 | 0.04 | 0.62 | **10.76** | 1.30 | 0.00 | 0.00 |
|  | IV | 4.92 | 0.00 | 0.00 | 14.07 | 0.00 | 61.83 | 0.15 | 0.16 | 0.13 | 1.77 | **15.64** | 1.14 | 0.17 | 0.02 |
| HoTaO_4_ | I | 9.16 | 1.62 | 2.19 | 0.00 | 14.54 | 63.66 | 0.17 | 0.30 | 0.28 | 0.30 | 0.62 | **4.77** | 1.64 | 0.75 |
|  | II | 5.22 | 0.05 | 0.00 | 14.16 | 0.00 | 61.77 | 1.49 | 2.35 | 1.85 | 2.43 | 2.24 | **3.48** | 3.58 | 1.38 |
|  | III | 4.84 | 0.73 | 6.41 | 0.00 | 11.74 | 63.56 | 0.00 | 0.02 | 0.04 | 0.11 | 1.85 | **10.29** | 0.41 | 0.00 |
|  | IV | 5.20 | 0.07 | 0.00 | 14.16 | 0.00 | 61.79 | 0.66 | 0.98 | 0.66 | 0.99 | 7.26 | **6.72** | 1.18 | 0.33 |
| YTaO_4_ | I | 9.07 | 1.68 | 2.19 | 0.00 | 14.39 | 63.63 | 0.23 | 0.51 | 0.58 | 0.77 | 0.91 | 1.23 | **3.97** | 0.84 |
|  | II | 5.14 | 0.06 | 0.00 | 14.40 | 0.00 | 61.83 | 1.00 | 1.49 | 1.19 | 1.65 | 1.53 | 2.39 | **8.14** | 1.18 |
|  | III | 7.43 | 1.31 | 3.31 | 0.00 | 13.58 | 63.70 | 0.10 | 0.07 | 0.02 | 0.06 | 0.06 | 0.39 | **6.36** | 3.61 |
|  | IV | 4.97 | 0.18 | 0.00 | 14.34 | 0.00 | 61.84 | 0.12 | 0.18 | 0.09 | 0.17 | 0.13 | 0.78 | **13.75** | 3.45 |
| ErTaO_4_ | I | 9.45 | 1.35 | 2.38 | 0.00 | 14.52 | 63.66 | 0.34 | 0.81 | 0.81 | 0.98 | 1.08 | 1.40 | 1.97 | **1.25** |
|  | II | 5.07 | 0.09 | 0.00 | 14.26 | 0.00 | 61.82 | 0.73 | 1.12 | 0.92 | 1.13 | 1.17 | 1.59 | 3.29 | **8.81** |
|  | III | 0.78 | 2.18 | 6.20 | 0.00 | 11.37 | 63.95 | 0.00 | 0.00 | 0.02 | 0.00 | 0.02 | 0.08 | 1.80 | **13.60** |
|  | IV | 4.85 | 0.24 | 0.00 | 14.33 | 0.00 | 61.86 | 0.11 | 0.13 | 0.07 | 0.11 | 0.06 | 0.17 | 2.05 | **16.02** |

**Table S3.** EPMA analysis of corrosion products of layered stack RETaO_4_ ceramic after CMAS corrosion at 1300^o^C for 75 h

| Composition (at.%) | | | | | | | | | | | | | | | |
| --- | --- | --- | --- | --- | --- | --- | --- | --- | --- | --- | --- | --- | --- | --- | --- |
|  |  | Ca | Mg | Al | Si | Ta | O | Nd | Sm | Eu | Gd | Dy | Ho | Y | Er |
| NdTaO_4_ | I | 9.51 | 1.91 | 1.98 | 0.00 | 14.55 | 63.52 | **1.88** | 2.89 | 1.81 | 0.74 | 0.27 | 0.57 | 0.22 | 0.15 |
|  | II | 5.52 | 0.05 | 0.00 | 13.64 | 0.00 | 61.61 | **8.19** | 3.97 | 2.38 | 1.84 | 0.62 | 1.37 | 0.56 | 0.25 |
|  | III | 9.11 | 2.32 | 1.36 | 0.00 | 14.22 | 63.41 | **6.02** | 1.27 | 0.81 | 0.39 | 0.23 | 0.66 | 0.09 | 0.11 |
|  | IV | 5.20 | 0.09 | 0.00 | 14.11 | 0.00 | 61.76 | **11.39** | 4.08 | 1.30 | 0.18 | 0.40 | 0.87 | 0.44 | 0.18 |
| SmTaO_4_ | I | 7.04 | 1.36 | 4.11 | 0.00 | 12.80 | 63.43 | 0.54 | **7.63** | 2.85 | 0.17 | 0.00 | 0.06 | 0.00 | 0.01 |
|  | II | 5.06 | 0.12 | 0.00 | 13.73 | 0.00 | 61.70 | 2.51 | **11.75** | 2.60 | 0.88 | 0.10 | 1.06 | 0.28 | 0.21 |
|  | III | 7.73 | 2.76 | 1.02 | 0.00 | 14.21 | 63.58 | 0.68 | **8.55** | 0.81 | 0.22 | 0.09 | 0.22 | 0.05 | 0.08 |
|  | IV | 5.03 | 0.06 | 0.00 | 13.68 | 0.00 | 61.73 | 2.58 | **8.60** | 3.14 | 1.59 | 0.75 | 1.80 | 0.68 | 0.36 |
| EuTaO_4_ | I | 9.41 | 1.77 | 2.19 | 0.00 | 14.41 | 63.52 | 1.09 | 2.17 | **3.56** | 0.82 | 0.06 | 0.54 | 0.25 | 0.21 |
|  | II | 4.83 | 0.11 | 0.01 | 13.57 | 0.00 | 61.73 | 0.54 | 2.02 | **15.43** | 0.66 | 0.76 | 0.14 | 0.12 | 0.08 |
|  | III | 4.44 | 1.06 | 5.18 | 0.00 | 11.63 | 63.54 | 0.08 | 0.64 | **12.32** | 0.92 | 0.00 | 0.13 | 0.00 | 0.06 |
|  | IV | 4.63 | 0.09 | 0.05 | 13.46 | 0.00 | 61.75 | 0.20 | 0.67 | **15.78** | 2.38 | 0.64 | 0.27 | 0.06 | 0.02 |
| GdTaO_4_ | I | 9.49 | 1.44 | 2.36 | 0.00 | 14.38 | 63.56 | 0.06 | 0.15 | 0.47 | **2.76** | 2.10 | 1.58 | 1.05 | 0.60 |
|  | II | 5.06 | 0.00 | 0.00 | 13.74 | 0.00 | 61.73 | 0.96 | 1.56 | 1.74 | **10.15** | 2.24 | 1.71 | 0.87 | 0.24 |
|  | III | 5.95 | 1.06 | 4.61 | 0.00 | 12.56 | 63.62 | 0.10 | 0.17 | 3.59 | **7.46** | 0.00 | 0.86 | 0.00 | 0.02 |
|  | IV | 5.12 | 0.00 | 0.00 | 13.79 | 0.00 | 61.74 | 1.22 | 1.63 | 1.47 | **8.12** | 2.58 | 2.35 | 1.38 | 0.60 |
| DyTaO_4_ | I | 8.92 | 1.66 | 2.51 | 0.00 | 14.39 | 63.64 | 0.46 | 0.81 | 0.93 | 1.02 | **2.66** | 1.83 | 0.77 | 0.40 |
|  | II | 5.23 | 0.00 | 0.00 | 14.08 | 0.00 | 61.78 | 0.84 | 1.22 | 1.14 | 1.47 | **8.79** | 3.80 | 1.16 | 0.49 |
|  | III | 8.05 | 1.66 | 2.50 | 0.00 | 14.02 | 63.67 | 0.24 | 0.38 | 0.33 | 1.82 | **6.22** | 0.80 | 0.25 | 0.06 |
|  | IV | 4.96 | 0.00 | 0.00 | 13.75 | 0.00 | 61.76 | 1.41 | 2.19 | 2.30 | 2.42 | **3.94** | 3.89 | 2.27 | 1.11 |
| HoTaO_4_ | I | 9.39 | 1.56 | 2.45 | 0.00 | 14.23 | 63.51 | 0.13 | 0.32 | 0.43 | 0.66 | 1.02 | **4.22** | 1.57 | 0.51 |
|  | II | 5.15 | 0.11 | 0.00 | 14.31 | 0.00 | 61.80 | 0.56 | 0.84 | 0.70 | 0.88 | 1.17 | **10.58** | 3.48 | 0.42 |
|  | III | 7.98 | 1.64 | 2.84 | 0.00 | 14.00 | 63.68 | 0.18 | 0.21 | 0.16 | 0.15 | 0.46 | **8.13** | 0.57 | 0.00 |
|  | IV | 5.05 | 0.17 | 0.00 | 14.23 | 0.00 | 61.79 | 0.12 | 0.18 | 0.10 | 0.09 | 0.40 | **11.52** | 6.19 | 0.16 |
| YTaO_4_ | I | 8.31 | 2.08 | 2.07 | 0.00 | 13.52 | 63.33 | 0.36 | 0.86 | 0.18 | 0.93 | 0.77 | 1.45 | **5.35** | 0.79 |
|  | II | 5.20 | 0.05 | 0.00 | 13.62 | 0.00 | 61.67 | 1.29 | 1.89 | 1.24 | 1.56 | 1.51 | 2.36 | **7.61** | 2.00 |
|  | III | 4.24 | 0.71 | 6.39 | 0.00 | 11.63 | 63.68 | 0.00 | 0.00 | 0.02 | 0.01 | 0.07 | 1.22 | **10.67** | 1.36 |
|  | IV | 4.85 | 0.16 | 0.00 | 14.14 | 0.00 | 61.83 | 0.14 | 0.13 | 0.08 | 0.09 | 0.18 | 2.27 | **15.42** | 0.71 |
| ErTaO_4_ | I | 9.00 | 1.92 | 1.49 | 0.00 | 14.32 | 63.54 | 0.35 | 0.52 | 0.33 | 0.49 | 0.76 | 1.27 | 1.43 | **4.58** |
|  | II | 5.08 | 0.06 | 0.00 | 14.35 | 0.00 | 61.85 | 1.37 | 1.99 | 1.44 | 1.70 | 1.76 | 2.30 | 3.42 | **4.68** |
|  | III | 6.29 | 0.98 | 3.53 | 0.00 | 13.14 | 63.81 | 0.04 | 0.06 | 0.04 | 0.06 | 0.06 | 0.02 | 1.73 | **10.24** |
|  | IV | 4.85 | 0.25 | 0.00 | 14.35 | 0.00 | 61.85 | 0.00 | 0.03 | 0.02 | 0.00 | 0.00 | 0.04 | 14.29 | **4.32** |

**Table S4.** EPMA analysis of corrosion products of layered stack RETaO_4_ ceramic after CMAS corrosion at 1300^o^C for 100 h
